# Supplementary material for: Insights into the key determinants of membrane protein topology enable the identification of new monotopic folds
Source: eLife. 2018 Aug 31;7:e40889. doi: 10.7554/eLife.40889 (PMC6133551; doi:10.7554/eLife.40889)
Supplement: Supplementary file 1. [file elife-40889-supp1.docx]

| **Supplementary Table 1 – Corresponding key residues in *C. jejuni* and *C. concisus* PglC** | | |
| --- | --- | --- |
| ***C. jejuni* PglC** | ***C. concisus* PglC (PDB ID** **5W7L)** | **Purpose in this study** |
| Glu3 | Arg3 | Cys substitution for bBBr crosslinking |
| Lys4 | Asn4 | Cys substitution for SCAM |
| Phe6 | Leu6 | Cys substitution for SCAM |
| Lys7 | Lys7 | Conserved residue |
| Arg8 | Arg8 | Conserved residue |
| Ser23 | Ser23 | Conserved residue |
| Pro24 | Pro24 | Conserved residue |
| Ile26 | Ile26 | Control Ala mutation for thermal shift assay |
| Leu27 | Ile27 | Control Ala mutation for thermal shift assay |
| Ser88 | Ser89 | Cys substitution for SCAM |
| Ile163 | Leu164 | Cys substitution for bBBr crosslinking |
| Asp168 | Asp169 | Conserved residue |
| Ser186 | Ser187 | Cys substitution for SCAM |
| Lys187 | Lys188 | Control Ala mutation for thermal shift assay |
| Glu188 | Glu189 | Control Ala mutation for thermal shift assay |
