# Supplementary material for: Insights into the key determinants of membrane protein topology enable the identification of new monotopic folds
Source: eLife. 2018 Aug 31;7:e40889. doi: 10.7554/eLife.40889 (PMC6133551; doi:10.7554/eLife.40889)
Supplement: Supplementary file 2. [file elife-40889-supp2.docx]

| **Supplementary Table 2 – Primers used for cloning and mutagenesis of PglC and LpxM variants** | |
| --- | --- |
| PglC_F_NdeI | 5’-AAAAAACATATGTATGAAAAAGTTTTTAAAAGAATTTTTG-3’ |
| PglC_R_XhoI | 5’-AAAAAACTCGAGGTTCTTGCCATTAAATTTCTCTG-3’ |
| K4C_F | 5’-GGAGATATACATATGTATGAATGCGTTTTTAAAAGAATTTTTG-3’ |
| K4C_R | 5’-CAAAAATTCTTTTAAAAACGCATTCATACATATGTATATCTCC-3’ |
| SUMO_K4C_F | 5’-GGTGGGATGTATGAATGCGTTTTTAAAAGAATTTTTG-3’ |
| SUMO_K4C_R | 5’-CAAAAATTCTTTTAAAAACGCATTCATACATCCCACC-3’ |
| F6C_F | 5’-CATATGTATGAAAAAGTTTGCAAAAGAATTTTTGATTTTATTTTAGC-3’ |
| F6C_R | 5’-GCTAAAATAAAATCAAAAATTCTTTTGCAAACTTTTTCATACATATG-3’ |
| SUMO_F6C_F | 5’-GGGATGTATGAAAAAGTTTGCAAAAGAATTTTTGATTTTATTTTAGC-3’ |
| SUMO_F6C_R | 5’-GCTAAAATAAAATCAAAAATTCTTTTGCAAACTTTTTCATACATCCC-3’ |
| S88C_F | 5’-GGAAAAATCGTTAGATGCTTAAGTTTGGATGAGCTTTTGC-3’ |
| S88C_R | 5’-GCAAAAGCTCATCCAAACTTAAGCATCTAACGATTTTTCC-3’ |
| S186_F | 5’-GGTTTTAAAACGAAGTGGGGTATGCAAAGAAGGCCATGTTAC-3’ |
| S186_R | 5’-GTAACATGGCCTTCTTTGCATACCCCACTTCGTTTTAAAACC-3’ |
| S23A_F | 5’-GCTTTAGTGCTTTTAGTGCTTTTTGCTCCGGTGATTTTAATC-3’ |
| S23A_R | 5’-GATTAAAATCACCGGAGCAAAAAGCACTAAAAGCACTAAAGC-3’ |
| P24A_F | 5’-GATTAAAATCACCGCAGAAAAAAGCACTAAAAGCACTAAAGC-3’ |
| P24A_R | 5’-GCTTTAGTGCTTTTAGTGCTTTTTGCTCCGGTGATTTTAATC-3’ |
| S23A/P24A_F | 5’-GCTTTAGTGCTTTTAGTGCTTTTTGCTGCGGTGATTTTAATC-3’ |
| S23A/P24A_R | 5’-GATTAAAATCACCGCAGCAAAAAGCACTAAAAGCACTAAAGC-3’ |
| I26A/L27A_F | 5’-GTGCTTTTTTCTCCGGTGGCTGCAATCACTGCTTTACTTTTAAAAATCAC-3’ |
| I26A/L27A_R | 5’-GTGATTTTTAAAAGTAAAGCAGTGATTGCAGCCACCGGAGAAAAAAGCAC-3’ |
| K187A/E188A_F | 5’-CGAAGTGGGGTAAGCGCAGCAGGCCATGTTACAACAGAG-3’ |
| K187A/E188A_R | 5’-CTCTGTTGTAACATGGCCTGCTGCGCTTACCCCACTTCG-3’ |
| K7A_F | 5’-GTATGAAAAAGTTTTTGCAAGAATTTTTGATTTTATTTTAGCTTTAGTGC-3’ |
| K7A_R | 5’-GCACTAAAGCTAAAATAAAATCAAAAATTCTTGCAAAAACTTTTTCATAC-3’ |
| K4C/K7A_F | 5’-GTATGAATGCGTTTTTGCAAGAATTTTTGATTTTATTTTAGCTTTAGTGC-3’ |
| K4C/K7A_R | 5’-GCACTAAAGCTAAAATAAAATCAAAAATTCTTGCAAAAACGCATTCATAC-3’ |
| F6C/K7A_F | 5’-GTATGAAAAAGTTTGTGCAAGAATTTTTGATTTTATTTTAGCTTTAGTGC-3’ |
| F6C/K7A_R | 5’-GCACTAAAGCTAAAATAAAATCAAAAATTCTTGCACAAACTTTTTCATAC-3’ |
| R8A_F | 5’-GTATGAAAAAGTTTTTAAAGCAATTTTTGATTTTATTTTAGCTTTAGTGC-3’ |
| R8A_R | 5’-GCACTAAAGCTAAAATAAAATCAAAAATTGCTTTAAAAACTTTTTCATAC-3’ |
| K4C/R8A_F | 5’-GTATGAATGCGTTTTTAAAGCAATTTTTGATTTTATTTTAGCTTTAGTGC-3’ |
| K4C/R8A_R | 5’-GCACTAAAGCTAAAATAAAATCAAAAATTGCTTTAAAAACGCATTCATAC-3’ |
| F6C/R8A_F | 5’-GTATGAAAAAGTTTGTAAAGCAATTTTTGATTTTATTTTAGCTTTAGTGC-3’ |
| F6C/R8A_R | 5’-GCACTAAAGCTAAAATAAAATCAAAAATTGCTTTACAAACTTTTTCATAC-3’ |
| K7A/R8A_F | 5’-GTATGAAAAAGTTTTTGCAGCAATTTTTGATTTTATTTTAGCTTTAGTGC-3’ |
| K7A/R8A_R | 5’-GCACTAAAGCTAAAATAAAATCAAAAATTGCTGCAAAAACTTTTTCATAC-3’ |
| K4C/K7A/R8A_F | 5’-GTATGAATGCGTTTTTGCAGCAATTTTTGATTTTATTTTAGCTTTAGTGC-3’ |
| K4C/K7A/R8A_R | 5’-GCACTAAAGCTAAAATAAAATCAAAAATTGCTGCAAAAACGCATTCATAC-3’ |
| F6C/K7A/R8A_F | 5’-CATATGTATGAAAAAGTTTGTGCAGCAATTTTTGATTTTATTTTAGC-3’ |
| F6C/K7A/R8A_R | 5’-GCTAAAATAAAATCAAAAATTGCTGCACAAACTTTTTCATACATATG-3’ |
| SUMO_E3_F | 5’-GGTGGGATGTATTGCAAAGTTTTTAAAAGAATTTTTGATTTTATTTTAGC-3’ |
| SUMO_E3_R | 5’-GCTAAAATAAAATCAAAAATTCTTTTAAAAACTTTGCAATACATCCCACC-3’ |
| I163C_F | 5’-GAACTTGATGTGTATTATGTGAAAAATTGCTCTTTTCTGCTTGATTTAAAAATC-3’ |
| I163C_R | 5’-GATTTTTAAATCAAGCAGAAAAGAGCAATTTTTCACATAATACACATCAAGTTC-3’ |
|  |  |
| LpxM_C73S_F | 5’-CAACCTGAGCCTGAGCTTCCCGGAACG-3’ |
| LpxM_C73S_R | 5’-CGTTCCGGGAAGCTCAGGCTCAGGTTG-3’ |
| LpxM_C240S_F | 5’-CTGATGAAGGTGAGCCGTGCGCGTGTG-3’ |
| LpxM_C240S_R | 5’-CACACGCGCACGGCTCACCTTCATCAG-3’ |
| LpxM_S8C_F | 5’-CCAAGAAAAACAACTGCGAATACATCCCGGAGTTC-3’ |
| LpxM_S8C_R | 5’-GAACTCCGGGATGTATTCGCAGTTGTTTTTCTTGG-3’ |
| LpxM_I11C_F | 5’-GAAACCAAGAAAAACAACAGCGAATACTGCCCGGAGTTC-3’ |
| LpxM_I11C_R | 5’-GAACTCCGGGCAGTATTCGCTGTTGTTTTTCTTGGTTTC-3’ |
| LpxM_S17C_F | 5’-GGAGTTCGACAAATGCTTTCGTCACCCGCG-3’ |
| LpxM_S17C_R | 5’-CGCGGGTGACGAAAGCATTTGTCGAACTCC-3’ |
| LpxM_M89C_F | 5’-GAACGTGAGGCGATTGTGGACGAGTGTTTTGCGACCG-3’ |
| LpxM_M89C_R | 5’-CGGTCGCAAAACACTCGTCCACAATCGCCTCACGTTC-3’ |
