# Supplementary material for: Insights into the key determinants of membrane protein topology enable the identification of new monotopic folds
Source: eLife. 2018 Aug 31;7:e40889. doi: 10.7554/eLife.40889 (PMC6133551; doi:10.7554/eLife.40889)
Supplement: Supplementary file 3. [file elife-40889-supp3.docx]

| **Supplementary Table 3 – Amino acid sequences of peptide folding** | |
| --- | --- |
| WT | N – VIDILGALFLLILTSPIIIATAIFI – C |
| S23A/P24A | N – VIDILGALFLLILTAAIIIATAIFI – C |
